# The study of indirect genetic effects reveals the nature of the home environment – a commentary on Mathias Valstad et al. (2026)

**DOI:** 10.1111/jcpp.70208

**Published:** 2026-07-29

**Authors:** Conor V. Dolan, Alexandra Starr

**Affiliations:** ^1^ Department of Biological Psychology Vrije Universiteit Amsterdam Amsterdam The Netherlands

**Keywords:** ADHD, genetics, parent–child interaction, environment

## Abstract

Valstad et al. (2026) presented important methodological advances in the study of indirect genetic effects in developmental psychology and psychiatry. By combining genetically informative family designs with measured genetic data, they addressed how sibling interaction and genetic nurture may contribute to developmental outcomes such as attention‐deficit/hyperactivity disorder (ADHD) and educational performance. Their work highlights the importance of disentangling direct genetic effects from indirect genetic effects that arise through family relationships and shared environments. We discuss the conceptual implications of these processes for developmental psychology and psychopathology, and emphasize the interpretational challenges associated with correlated genetic and environmental influences. We note that heterogeneity in developmental processes may complicate statistical modeling approaches designed to estimate indirect genetic effects.

Valstad et al. (2026) presented important methodological advances for the study of indirect genetic effects in developmental psychology and psychiatry. By combining genetically informative family designs with measured genetic data, they addressed how sibling interaction and genetic nurture may contribute to developmental outcomes such as attention‐deficit/hyperactivity disorder (ADHD) and educational performance. Their work highlights the importance of disentangling direct genetic effects from indirect genetic effects that arise through family relationships and shared environments. In this commentary, we discuss the conceptual implications of these processes for developmental psychology and psychopathology, emphasize the interpretational challenges associated with correlated genetic and environmental influences, and consider how heterogeneity in developmental processes may complicate statistical modeling approaches designed to estimate indirect genetic effects.

The authors combined genetically informative family designs and observed genetic information to address processes that potentially give rise to correlation between genetic and environmental predictors of phenotypes (henceforth *corGE*), such as ADHD and educational performance. The two key processes considered are sibling interaction and genetic nurture. Sibling interaction refers to the reciprocal influence among siblings. This occurs when the heritable behaviors of siblings contribute to each other's effective environment. For example, ADHD‐related behaviors such as impulsivity or inattention of one sibling may shape the home environment of another sibling through increased disruption of shared routines and social interactions, thereby influencing the co‐sibling's behavioral outcomes. So, the term describes reciprocal social influences between siblings, rather than an interaction in the statistical sense (e.g. genotype–environment interaction). Genetic nurture refers to environmental effects on offspring that stem from parents' heritable behavior in addition to genes transmitted to the child. For example, parents' genetically influenced reading‐related traits may shape shared reading practices or access to books, influencing children's reading development independently of genetic transmission. The word ‘nurture’ is possibly misleading as it suggests beneficial parental influence, but it is possible that the influence is detrimental, for example, in the development of psychopathological traits or conditions. As Valstad et al. (2026) explain, direct genetic effects concern the effects of genes of individuals on the phenotypes of these individuals. This concerns the usual interpretation of genetic effects, as in the statement ‘the heritability of ADHD in children is about .75’. Indirect effects arise through sibling interaction and genetic nurture, where the genes of (close) relatives of individuals (probands) influence the effective environment of the individuals. The corGE arises because close relatives share alleles (identically by descent). To understand the origin of corGE, it is helpful to interpret these processes in terms of mediation. Specifically, in the case of genetic nurture: the effects of the genes of parents (close relatives) on the effective environment of the offspring (probands) are mediated by heritable parenting behaviors. The alleles underlying variation in heritable parental behavior, by Mendel's law of segregation, are transmitted to the offspring. If these alleles contribute to variation in the offspring outcome, corGE arises. The primary example of this is offspring educational performance (Wang et al., 2021), but it is plausible in developmental psychopathology.

Valstad et al. (2026) considered sibling interaction in a random effects regression model, and combined sibling effects and genetic nurture in a fixed effects structural equation model. They argued rightly that the processes of sibling interaction and genetic nurture are not mutually exclusive, and that it is important to resolve these processes in the study of human psychological development. In a nutshell, the interpretative issue is that a statistical method designed to detect indirect genetic effects (in the presence of direct genetic effects) may produce positive results that are hard to interpret, as these may be consistent with multiple underlying mechanisms. Bernardo, Pahnke, van Bergen, and Dolan (2026) showed that indirect genetic effects attributable to sibling interaction can give rise to results that suggest the presence of genetic nurture, and vice versa. While Valstad et al. (2026) and Bernardo et al. (2026) focus on sibling interaction and genetic nurture, there are other sources of corGE, such as subtle population stratification or dynastic and extended family effects that shape offspring environments across generations (Nivard et al., 2024).

The current interest in indirect genetic effects and corGE processes (see the references in Valstad et al., 2026) is partly attributable to the opportunities afforded by measured genetic variables (i.e. single nucleotide polymorphisms) to study these processes. The statistical methods, as developed and applied by Valstad et al. (2026), hinge on the presence of measured genetic information collected in genetically informative designs, such as twin and family studies. While the ‘GWAS revolution’ marked a move away from twin and family studies, it is well established that such study designs are useful in the GWAS setting (Howe et al., 2022; Nivard et al., 2024; Visscher, Andrew, & Nyholt, 2008), and that they are essential to address sibling interaction and genetic nurture. We note that Lindon Eaves, about 50 years ago, presented and formalized these processes in the classical twin design (sibling interaction) and the nuclear twin family design (genetic nurture, but Eaves used the term cultural transmission; see Eaves, Last, Martin, & Jinks, 1977).

The importance of the simultaneous study of direct and indirect genetic effects, notably in developmental psychology, bears reiteration. From the point of view of parental influence on offspring educational performance, this is important because it tells us that the home environments that parents create are not necessarily strictly environmental but that they may harbor a genetic component. This has a bearing on the interpretation of phenotypic associations between a given environmental measure and offspring outcomes, such as ADHD or educational performance. First, what should we consider to be actually environmental in the study of environmental determinants of educational performance of children? Second, is the association between, say, the number of books and children's interest in reading in part attributable to genetic confounding stemming from direct genetic effects, that is alleles that predispose parents and their offspring to like reading? Establishing that the association is present while taking into account direct genetic effects is necessary (but not sufficient) for the interpretation of the association as causal (‘book in the home causes interest in reading’). Indirect genetic effects in the context of educational performance, resulting in positive corGE, are well established (Wang et al., 2021). The positive corGE implies that the offspring of highly educated parents are at a double advantage: they inherit alleles and are raised in home environments that are conducive to good educational performance. Looking beyond the cognitive developmental implications, we note that this double advantage is a source of educational inequity, which is difficult to redress in meritocratic societies.

In the context of developmental psychopathology, corGE is important simply because the correlation between genetic and environmental factors will, ceteris paribus, affect the prevalence of ADHD. The magnitude of the correlation does not have to be large. For instance, in the case of a heritability of .75 and a prevalence of .05 (both typical of ADHD) given zero corGE, the prevalence will increase to about .07 given a positive correlation of .12 and will decrease to about .03 given a negative correlation of −.12. Insight into the processes that give rise to corGE in psychopathology raises our awareness concerning the complex interrelatedness of general home environment, which includes the interactions among family members, and the genetic predisposition to develop a psychopathological condition. At the phenotypic level, parenting and home environment are well known to be associated with ADHD (Claussen et al., 2024). However, relatively little is known about the contribution of genetic confounding (in addition to the purported causal influence), attributable to the direct genetic effect of alleles shared by parents and offspring, and about the interdependence of genetic predisposition and the home environment due to the indirect effects, as studied by Valstad et al. (2026; see also Eilertsen et al., 2021). These considerations also raise broader methodological questions concerning the assumptions underlying statistical models used to estimate indirect genetic effects and corGE processes.

More generally, it is important to note that the statistical methods (including those of Valstad et al., 2026) are based on the assumption of homogeneity of processes. That is, the processes, as analyzed statistically in general population samples, are assumed to hold generally in the relevant population. This seems trivial, but homogeneity is a strong assumption.

First, one obvious source of heterogeneity in developmental studies is the actual age of children. It seems reasonable to suppose that in a developmental study the magnitude of indirect genetic effects (and any resulting corGE), will depend on children's age. Considering genetic nurture, the intensity and the nature of parental behaviors relevant to educational performance or psychopathology are likely to depend on the age of the child. Valstad et al. (2026) take this into account by stratifying by age in their study of ADHD and educational performance. However, their SEM‐PGI model can be extended to include age as a continuously distributed moderator, as moderation modeling is well established in structural equation modeling (SEM) and in regression modeling. Age moderation is also a concern in any statistical model that includes polygenic indices, because the calculation of polygenic indices is based on the effect sizes of associated genetic variants, which are often established in adult samples. Prior to adulthood, these effect sizes are likely to be subject to age moderation (i.e. age × gene interaction).

Second, other forms of heterogeneity present problems that are less tractable than age moderation. For instance, the direction of the influence of one family member on the other may vary from family to family. In the study of children, it seems reasonable to assume that parental behavior (genetic nurture) will influence the environment relevant to the educational performance of children. However, in the case of externalizing psychopathology, for instance, parent–child influences may be bidirectional. Indirect genetic influences involving offspring disruptive behavior may contribute to the effective home environment as experienced by the parents.

Third, heterogeneity may characterize the extremes of phenotypic distributions. The well‐established *massive* polygenicity of psychopathology guarantees a continuous and approximately normal underlying genetic liability. However, just as the magnitude of main effects of genes on, say, depression may be moderated by age or environmental stressors, such as adverse childhood experiences (i.e. the diathesis‐stress model), the very nature of interplay among genetic disposition and the environment may be subject to moderation as well. The nature of the moderation itself may depend on the severity of the psychological disorder. Specifically, in the case of adults, it seems plausible that the manner in which a depressed individual interacts with their environment may depend on the severity and the persistence of their depression. The estimates of indirect genetic effects and corGE derived from population‐based models thus may reflect an average process, which does not rule out qualitative variation in the processes operating at the extreme ends of the phenotypic liability continuum.

In conclusion, the work by Valstad et al. (2026) underscores the importance of disentangling direct and indirect genetic effects in genetically informative studies. Understanding the mechanisms that give rise to correlations between genetic and environmental influences – whether through genetic nurture, sibling interaction or any other source of corGE – is essential for the interpretation of associations between family environments and developmental outcomes. As such, we emphasize that this work, notwithstanding its emphasis on and use of extensive genetic data, shines a light on the role of the behaviors of family members as contributors to the (developmental) home environment.

## Ethical considerations

Ethical approval is not applicable to this Commentary Article.

## Data Availability

Data sharing not applicable to this article as no datasets were generated or analyzed during the current study.
